# A Complex Role of Herpes Viruses in the Disease Process of Multiple Sclerosis

**DOI:** 10.1371/journal.pone.0105434

**Published:** 2014-08-22

**Authors:** Simone C. Wuest, Ina Mexhitaj, Noo Ri Chai, Elena Romm, Joerg Scheffel, Biying Xu, Kelly Lane, Tianxia Wu, Bibiana Bielekova

**Affiliations:** 1 Neuroimmunological Diseases Unit, Neuroimmunology Branch, National Institute of Neurological Disorders and Stroke, National Institutes of Health, Bethesda, Maryland, United States of America; 2 Molecular Immunology Section, Laboratory of Molecular Immunogenetics, National Institute of Arthritis and Musculoskeletal and Skin Diseases, National Institutes of Health, Bethesda, Maryland, United States of America; 3 Imaging Probe Development Center, National Heart, Lung, and Blood Institute, National Institutes of Health, Bethesda, Maryland, United States of America; 4 Clinical Neurosciences Program, National Institute of Neurological Disorders and Stroke, National Institutes of Health, Bethesda, Maryland, United States of America; Julius-Maximilians-Universität Würzburg, Germany

## Abstract

Multiple sclerosis (MS) is a chronic inflammatory disorder of the central nervous system (CNS). Neither the antigenic target(s) nor the cell population(s) responsible for CNS tissue destruction in MS have been fully defined. The objective of this study was to simultaneously determine the antigen (Ag)-specificity and phenotype of un-manipulated intrathecal CD4^+^ and CD8^+^ T cells of patients with relapsing-remitting and progressive MS compared to subjects with other inflammatory neurological diseases. We applied a novel Ag-recognition assay based on co-cultures of freshly obtained cerebrospinal fluid T cells and autologous dendritic cells pre-loaded with complex candidate Ag's. We observed comparably low T cell responses to complex auto-Ag's including human myelin, brain homogenate, and cell lysates of apoptotically modified oligodendroglial and neuronal cells in all cohorts and both compartments. Conversely, we detected a strong intrathecal enrichment of Epstein-Barr virus- and human herpes virus 6-specific (but not cytomegalovirus-specific) reactivities of the Th1-phenotype throughout all patients. Qualitatively, the intrathecal enrichment of herpes virus reactivities was more pronounced in MS patients. This enrichment was completely reversed by long-term treatment with the IL-2 modulating antibody daclizumab, which strongly inhibits MS disease activity. Finally, we observed a striking discrepancy between diminished intrathecal T cell proliferation and enhanced cytokine production of herpes virus-specific T cells among progressive MS patients, consistent with the phenotype of terminally differentiated cells. The data suggest that intrathecal administration of novel therapeutic agents targeting immune cells outside of the proliferation cycle may be necessary to effectively eliminate intrathecal inflammation in progressive MS.

## Introduction

Multiple sclerosis (MS), the most prevalent neuroimmunological disorder in young adults, is primarily characterized by demyelination and axonal loss and leads to severe disability over time [1]. Analogous to experimental autoimmune encephalomyelitis (EAE), which can be induced in genetically susceptible animals through immunization with varied myelin epitopes, MS has been considered to be mediated by CD4^+^ Th1/Th17 cells that specifically target myelin. Yet, despite significant efforts to verify myelin target(s), to identify new antigens (Ag's) or to define pathogenic immune cell types, we have to conclude that mechanisms by which the immune system mediates tissue destruction of the central nervous system (CNS) in MS remain unclear.

The majority of published studies addressing Ag-specificity of T cells in MS derived both T cells and Ag-presenting cells (APCs) from peripheral blood mononuclear cells (PBMCs) [2]–[4]. Due to the limited number of professional APCs in the blood, such as dendritic cells (DCs), most of the published studies utilized (myelin-derived) peptides loaded exogenously onto surface-expressed major histocompatibility complex (MHC) molecules. This greatly restricted the amount of epitopes that could be tested and eliminated any post-translational modifications that might be crucial to the immunogenicity of auto-Ag's [5], [6]. Furthermore, because peptides bind with different affinities to various MHC alleles, observed differences between patient and control populations may simply reflect variances in the MHC composition, as the MHC locus represents the strongest regions of genetic susceptibility to MS [7], [8]. Finally, due to differential peptide-length requirements for MHC class I versus MHC class II exogenous loading, such assays could only test CD4^+^ or CD8^+^ T cell reactivity individually, but not in parallel. Even when studies utilized complex Ag's (such as myelin or its proteins), the perceived difference in the reactivities to such Ag's between MS patients and controls could have originated in APC differences, such as their frequency or activation status. Therefore, to unequivocally demonstrate a difference in the T cell compartment, one has to assure that the concentration and activation status of APCs is comparable between cohorts. This is not trivial, as it requires purification of T cells and utilization of exogenous APCs.

Perhaps the most urgent question is whether or not peripheral blood reliably reflects what is happening in the intrathecal compartment. Several publications indicate that this may not be the case: 1. multiple studies of soluble inflammatory biomarkers observed no or even opposite correlations between blood and cerebrospinal fluid (CSF) [9]–[11]; 2. sequencing of B cell receptors (BCRs) derived from paired blood and CSF samples demonstrated on average less than 5% overlap between the two compartments [12]; and 3. expansion of autoimmune T cells induced by CNS injuries is detectable in the blood months after the injury, whereas in acute phases of experimentally induced stroke the precursor frequency of brain-specific T cells is actually decreased in comparison to control animals, because these cells are preferentially recruited to the injured tissue [13], [14]. Therefore, it appears that blood and CNS comprise distinct immunological compartments. Preferential recruitment and retention of pathogenic cells in the CNS tissue at the time of acute or ongoing CNS inflammation may account for their depleted levels in the blood [15].

Though CNS tissue is generally inaccessible in living subjects, several investigators analyzed the Ag-specificity of CSF cells. These rare cases, while extremely valuable, are subject to the same technical caveats associated with blood studies. Furthermore, due to limited quantities of CSF T cells, most of the studies had to rely on the pre-expansion of CSF T cells by some type of polyclonal stimulus, which may alter the precursor frequency or phenotype of Ag-specific T cells in an unpredictable manner.

Therefore, we decided to revisit the issue of T cell Ag-specificity in MS, building on past experiences and capitalizing on recent methodological advancements. We wanted to develop an assay that would address above-mentioned drawbacks: 1. we desired to study Ag-specificity of CD4^+^ and CD8^+^ T cells in parallel, because this is how they are activated under physiological conditions (e.g. infections). 2. In order to mimic physiological situations, we employed autologous monocyte-derived DCs, which process and load exogenous complex Ag's to MHC-II and, via cross-presentation, to MHC-I molecules. DCs loaded with complex Ag's *in vitro* select immunodominant epitopes identical to those selected upon encountering identical Ag's *in vivo*, according to the MHC background of the individual. 3. The notion that pathogenic responses in MS may target non-myelin Ag's has not been excluded. Therefore, we desired to test T cell reactivity to candidate Ag's other than myelin, such as human brain homogenate (BH) or infectious agents (i.e. human herpes virus-6 (HHV6) and Epstein-Barr virus (EBV)) [16]–[18]. Finally, novel epitopes may also arise as non-physiological post-translational modifications during pathological processes, e.g. oxidative or genotoxic stress. In order to study all of these possibilities, we carefully selected a set of complex Ag's utilized in our current study. 4. The dual roles of Ag-specific T cells as detrimental and beneficial in the MS disease course prompted us to evaluate Ag-specific CD4^+^ and CD8^+^ T cell cytokine profiles. 5. To assure that responses attributed to MS were disease-specific, we employed patients with other inflammatory neurological disorders (OIND) as a control group. 6. To assess a relationship between the Ag-specificity and the disease process of MS, we also included a group of MS patients on long-term daclizumab (DAC HYP) therapy, who lacked disease activity by clinical and magnetic resonance imaging (MRI) criteria. 7. To minimize the influence of biases, all subjects were studied prospectively in a blinded fashion using written standard operating procedures (SOPs). Unblinding occurred only after reaching a pre-determined number of patients (n = 100) in accordance with recommendations for performance of biomedical research studies [19].

## Materials and Methods

### Study subjects

The study was approved by the NIH Institutional Review Board and all patients provided written consent. Analyses were performed in accordance with institutional guidelines of the NIH. Patients were prospectively recruited and assigned alphanumeric codes. The study was terminated after reaching a pre-determined sample size (n = 100). Based on prior recruitment experience, we estimated a relatively equal representation of selected diagnostic categories, which was confirmed after unblinding.

Un-treated cohorts consisted of 21 primary-progressive (PPMS), 20 secondary-progressive (SPMS) and 21 relapsing-remitting (RRMS) MS patients. The un-treated control group was comprised of 19 patients with OIND. In addition to the un-treated RRMS cohort, 19 RRMS patients received long-term treatment (>36 months) with DAC HYP (provided by AbbVie Biotherapeutics and Biogen Idec), a version of daclizumab under clinical trial evaluation (Clinicaltrials.gov identifier NCT01143441). All biological samples were collected, processed and analyzed in a blinded fashion using SOPs. Patients' demographic data are provided in **Table 1**.

**Table 1:** Patients' demographics and clinical characteristics.

	Controls, n = 19	Relapsing-Remitting MS (RR), n = 40		Progressive MS (Prog.), n = 41	
	OIND, un-treated^a^	RRMS, treated^b^	RRMS, un-treated	SPMS, un-treated	PPMS, un-treated
**Gender [Female/Male]**	19 [8/11]	19 [8/11]	21 [18/3]	20 [11/9]	21 [12/9]
**Median age [Range]**	57.0 [22–74]	44.0 [28–61]	37.0 [25–65]	58.0 [43–66]	53.0 [43–65]
**Median EDSS [Range]**	3.5 [0.0–7.0]^c^	1.5 [1.0–5.0]	1.5 [0.0–6.5]	6.5 [2.5–6.5]	6.0 [2.0–6.5]
**Race**	16 Cau/ 2 AA/ 1 A	18 Cau/ 1 A	14 Cau/ 5 AA/ 1 MR/ 1 Unkn	17 Cau/ 2 AA/ 1 MR	20 Cau/ 1 AA

a Diagnoses: Cryptococcal meningitis (7), recurrent meningitis (1), systemic lupus erythematosus (1), cyclic fever (2), neuromyelitis optica (1), autoimmune lymphoproliferative syndrome-like disorder with CELs (1), persistently enhancing C-spine lesions (1), leukodystrophy-like disorder (1), CLIPPERS (Chronic Lymphocytic Inflammation with Pontine Perivascular Enhancement Responsive to Steroids) (1), unclear diagnosis (3).

b Long-term treatment with DAC HYP.

c EDSS (Expanded Disability Status Scale) not available for each patient.

A: Asian, AA: African American, Cau: Caucasian, MR: Mixed race, Unkn: Unknown.

### CSF sample collection and processing

Lumbar punctures (LPs) were performed between the hours of 9 am and 12 pm. An average volume of 20 ml CSF was collected and spun immediately (1200 rpm, 10 min., 4°C). The CSF supernatant was removed from the cell pellet and stored on ice. CSF cells were re-suspended in x-vivo media (Lonza), counted using a Neubauer hemocytometer and distributed among different assays.

### Polyclonal expansion of CSF T cells

Freshly obtained CSF cells were seeded in a 96 round bottom well plate and polyclonally stimulated with anti-CD3/CD28 Dynabeads (Invitrogen) at a 1∶1 bead to T cell ratio in 100 µl of T cell media (IMDM, Penicillin, Streptomycin, Gentamicin, L-Glutamine, and 10% human serum). Using a magnet, CSF cells were depleted of Dynabeads 72 hours later and subsequently cultured in IL-2 enriched T cell media (final IL-2 concentration 30 IU/ml; National Cancer Institute (NCI) Frederick). After 10–14 days cells were washed with x-vivo media, counted and frozen in liquid nitrogen until usage.

### EBV-transformation of intrathecal B cells

One day prior to the LP cultured CD40L-expressing NIH3T3 cells were irradiated at 6000 RAD, seeded in a 384 well plate at 5×10^3^ cells per 100 µl of B cell media (RPMI, Penicillin, Streptomycin, L-Glutamine, and 15% fetal bovine serum) and incubated overnight at 37°C, 5% CO_2_. On the day of LP 1×10^4^ of fresh CSF cells were re-suspended in a mix of EBV-containing B95.8 cell supernatant, CpG ODN 2006 (InvivoGen), IFN-γ (PeproTech) and Cyclosporine A (Sigma Aldrich) and added to NIH3T3 cells. When B cell clusters were visible and cells were growing, they were split and transferred to bigger plates until they reached sufficient numbers for preparation of cell lysates.

### EBV-transformation of peripheral B cells

1×10^7^ PBMCs were cultured in equal volumes of B cell media and EBV-containing B95.8 cell supernatant. After subsequent addition of the anti-CD3 antibody (Ab) OKT3 (NCI), cells were cultured at 37°C, 5% CO_2_ without changing media for six days. After 7-10 days B cells showing initial cell clusters were grown to higher cell numbers for following lysis and/or freezing.

### Preparation of antigens

Ag's for the entire project were prepared at once, aliquoted in single-use vials, frozen at -80°C and thawed minutes prior to loading of DCs. An overview of the candidate Ag's is provided in **Table 2**.

**Table 2:** Classification of candidate Ag's.

Condition	Abbreviation	Concentration (µg/ml)	Relevance for MS
No antigen	No Ag		Negative control; background proliferation and cytokine secretion
Brain homogenate	BH	5.0	Auto-Ag; derived from snap-frozen MS brain <8 hrs post-mortem
Human myelin	Myelin	2.5	Auto-Ag; derived from snap-frozen MS brain <8 hrs post-mortem
Differentiated MO3.13 cell lysate	MO313	1.0	Auto-Ag; human oligodendroglial cell line post-exposure to H_2_O_2_
SK-N-SH cell lysate	SKNSH	5.0	Auto-Ag; human neuronal cell line post-exposure to UVA light
EBV viral lysate	EBV	1.0	Foreign Ag; epidemiologically linked to MS
HHV-6 viral lysate	HHV6	1.0	Foreign Ag; linked to MS by previous observational studies
CMV viral lysate	CMV	1.0	Foreign control Ag
EBNA-1_458-641_ peptide	EBNA	1.0	Immunodominant EBV protein previously linked to MS
CSF EBV-B cell lysate	CSF Bc	5.0	Combination of EBV and auto-Ag; to test anti-idiotypic interactions
Peripheral EBV-B cell lysate	Periph Bc	5.0	Combination of EBV and auto-Ag; to test anti-idiotypic interactions

Optimal concentrations of candidate Ag's were selected based on pilot experiments.

### Whole brain homogenate and myelin

BH and myelin were isolated from a post-mortem brain of a patient with pathologically confirmed PPMS. Brain tissue was collected within eight hours of death, immediately snap frozen and kept at −80°C for less than one year. BH and myelin isolations were processed according to previously described methods [20], [21]. Protein contents were determined using a BCA protein assay (Pierce) and adjusted to 1 µg/ml with 1x PBS.

### Lysates from modified oligodendroglial and neuronal cells

In preliminary experiments, intrathecal T cell and Ab reactivities of MS patients were assessed in a small, unblinded cohort in response to four human cell lines exposed to various pro-apoptotic stimuli. Undifferentiated MO3.13 oligodendroglial precursors, PMA-differentiated MO3.13 oligodendroglial cells, SKNSH neuronal cells and CCF-STTG1 astroglial cells were exposed to UVA light (as representative of genotoxic stress), H_2_O_2_ (oxidative stress), heat shock and hypoxia. Cell lysates were prepared after cells started to develop phenotypical signs of apoptosis (membrane blebbing, picnotic nuclei, positive Annexin V staining). Out of these 16 conditions, differentiated MO3.13 cells exposed to oxidative stress and SKNSH cells exposed to UVA light were selected as most immunodominant conditions for further testing.

Undifferentiated MO3.13 and neuronal SKNSH cells were cultured until 80–100% confluency. Differentiation of MO3.13 cells was initiated by addition of 100 nM PMA (Sigma Aldrich) to serum-free media. After three days cells were exposed to 450 µM H_2_O_2_. For induction of genotoxic effects, SKNSH cells were exposed to UVA-light (Black Ray XX-15 BLB, Upland CA, 15 Watt, 115 V, 365 nm). After 15 hours, MO3.13 and SKNSH cells were harvested and lysed by five freezing/thawing cycles. Crude lysates were spun (14,000 rpm, 10 min., at 4°C). DNA precipitations were digested with rDNase I (Ambion; 25 min., 37°C) which was inactivated by EDTA (final concentration 5 mM) and heat incubation (10 min., 75°C). After spinning (14,000 rpm, 4 min., 4°C) pellets were re-suspended in 1x PBS. The protein content was determined using a BCA protein assay and the concentration was re-adjusted to 1 µg/ml with 1x PBS.

### Expression and purification of recombinant EBNA-1_458-641_

The expression and purification of recombinant EBNA-1_458-641_ (EBV nuclear antigen-1) were performed as described previously [22]. The final concentration was adjusted to 1 µg/ml with 1x PBS. A glycerol stock containing transformed, EBNA-1_458-641_ expressing *Escherichia coli* BL21 (DE3) pLysS cells was a kind gift from Dr. Jan Lünemann (University of Zurich, Switzerland).

### Lysates from EBV-transformed peripheral and intrathecal B cells

EBV-transformed B cells were collected, washed and counted. 1×10^7^ B cells were re-suspended in 1 ml of 20 mM Tris-buffer containing protease inhibitors (Roche) and lysed by five freezing/thawing cycles. DNA digestion was processed as described above. Protein contents were determined using a BCA protein assay and adjusted to 1 µg/ml with 1xPBS.

### Isolation of PBMCs and generation of DCs

120 ml of blood or lymphocytapheresis samples were collected eight days before the LP. Isolation of PBMCs and generation of DCs were performed as described previously [23]. Immature DCs were co-incubated overnight with Ag's and subsequently stimulated for 48 hours. The DC phenotype was characterized by flow cytometric staining for MHC-II, CD11c, CD25, CD80 and CD83 (all BD Biosciences and eBioscience).

### Co-culture of Ag-loaded mature DCs and T cells

At the day of LP peripheral T cells were purified from PBMCs by negative selection (Miltenyi Biotech). Peripheral and CSF T cells were then cultured in round bottom 96 well plates with autologous Ag-loaded mature DCs (3×10^3^ mDCs: 3×10^3^ T cells) in a total volume of 100 µl x-vivo media at 37°C, 5% CO_2_. After seven days, T cells were re-stimulated overnight for the intracellular cytokine staining (ICCS) with twice as much fresh Ag-loaded mDCs as used for the first co-culture in presence of Brefeldin A and monensin (eBioscience).

### Flow cytometric analysis of surface markers and intracellular cytokines

T cells were analyzed for surface markers CD3 (UCHT1), CD4 (RPA-T4), and CD8 (SK1), and intracellular expression of IL-2 (MQ1-17H12), BDNF (35909), GM-CSF (GM2F3), IL-17 (eBio64DEC17), TNF-α (MAb11) and IFN-γ (B27; BD Biosciences, eBioscience and R&D Systems). Gated on CD3^+^CD4^+^ and CD3^+^CD8^+^ T cells, intracellular cytokine secretion was detected utilizing proper isotype controls. Data were analyzed with BD FACS Diva 6.1 (BD Biosciences).

Numbers of cytokine-producing cells were determined by normalization with fluorescent beads. For inter-patient comparison we standardized the number of cytokine positive events (i.e. IFN-γ^+^, TNF-α^+^, and double positive events) to 1000 beads each. Ratios were calculated from intrathecal to peripheral T cell reactivities in response to Ag's. Ratios greater than one indicate enrichment of Ag-specific T cell events in the intrathecal compartment; ratios lower than one represent less pronounced intrathecal T cell responses.

Mean fluorescence intensities (MFIs) of cytokine-secreting CD4^+^ and CD8^+^ T cells were determined by subtraction of the respective isotype controls. Negative values were adjusted to zero. MFI ratios of intrathecal to peripheral CD4^+^ and CD8^+^ T cells were calculated. Ratios lower than one indicate increased cytokine production of peripheral T cells, whereas levels greater than one emphasize intrathecal cytokine production.

### Inflammation human membrane antibody array

CSF T cell supernatants were collected seven days after co-culture and stored at −20°C. Membranes (Abcam) and samples (1 ml of combined supernatants) were prepared according to the manufacturer's instructions. Membranes were scanned using the Odyssey SA and densitometrically analyzed (Image Studio software; LI-COR). After subtraction of negative controls, samples were standardized to positive control values and multiplied by 100 (arbitrary unit).

### Statistical analysis

A paired t-test was performed to evaluate the difference between peripheral and intrathecal Ag-specific T cell proliferation (dependent variable), for each cohort (OIND, Prog. and RR) and condition (independent variable). A two sample t-test was used to test the difference between Ag-specific T cell proliferation in the un-treated and DAC HYP-treated RRMS cohort. Peripheral and intrathecal T cells were analyzed separately.

ANOVA was applied to raw MFI data to assess the effect of the condition on TNF-α and IFN-γ secretion by CD4^+^ and CD8^+^ T cells for each cohort. Raw MFI data from peripheral and intrathecal T cells were analyzed separately. Dunnett's method was used for the post-hoc test with the No Ag condition as a control. The above ANOVA was also applied to the MFI ratios of intrathecal to peripheral TNF-α^+^ and IFN-γ^+^ CD4^+^ and CD8^+^ T cells. In the calculation of ratios, the zero values were treated as missing values.

Box-Cox transformation was applied to Ag-specific T cell proliferation data and their ratios (natural logarithm), MFI raw data (quadratic root), and MFI ratios (natural logarithm). The Shapiro-Wilk test was performed to test for normality. All above statistical analyses were performed using SAS version 9.2 with p≤0.05 considered a significant level.

## Results

### Development of the CSF Ag-specificity assay

**Figure 1:**
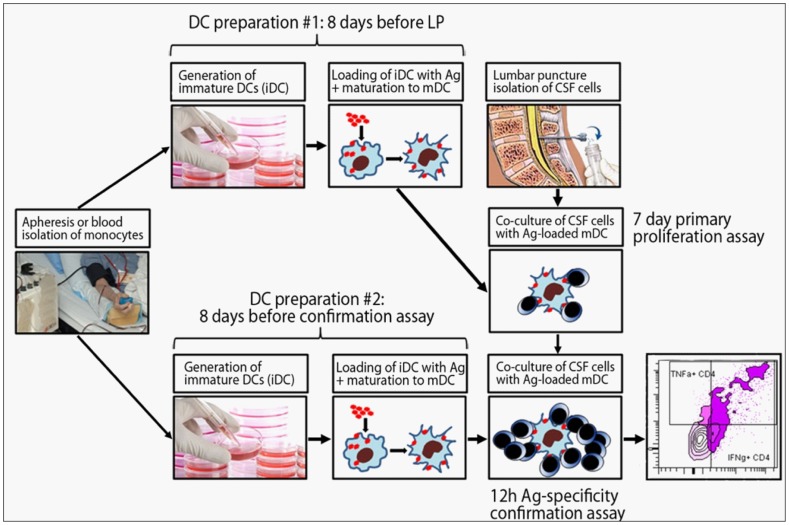
CSF T cell recognition and confirmation assay. PBMCs were obtained from apheresis samples or blood, and monocytes were isolated via positive selection with CD14^+^ magnetic beads. For the primary proliferation assay, monocytes were differentiated into immature dendritic cells (iDC). On the sixth day of culture, iDCs were loaded with selected Ag's and stimulation cocktail was added to induce DC maturation. 48 hours later, mature DCs (mDC) were co-cultured with peripheral and intrathecal T cells. After seven days of proliferation, cultured T cells were re-stimulated overnight with newly differentiated, identically loaded mDCs. Ag-specificity was detected by flow cytometric analysis of cytokine-producing CD4^+^ and CD8^+^ T cells.

Our aim was to develop an assay to quantify CD4^+^ and CD8^+^ T cell reactivities to complex Ag's potentially targeted by pathogenic immune responses (**Figure 1**).

**Figure 2:**
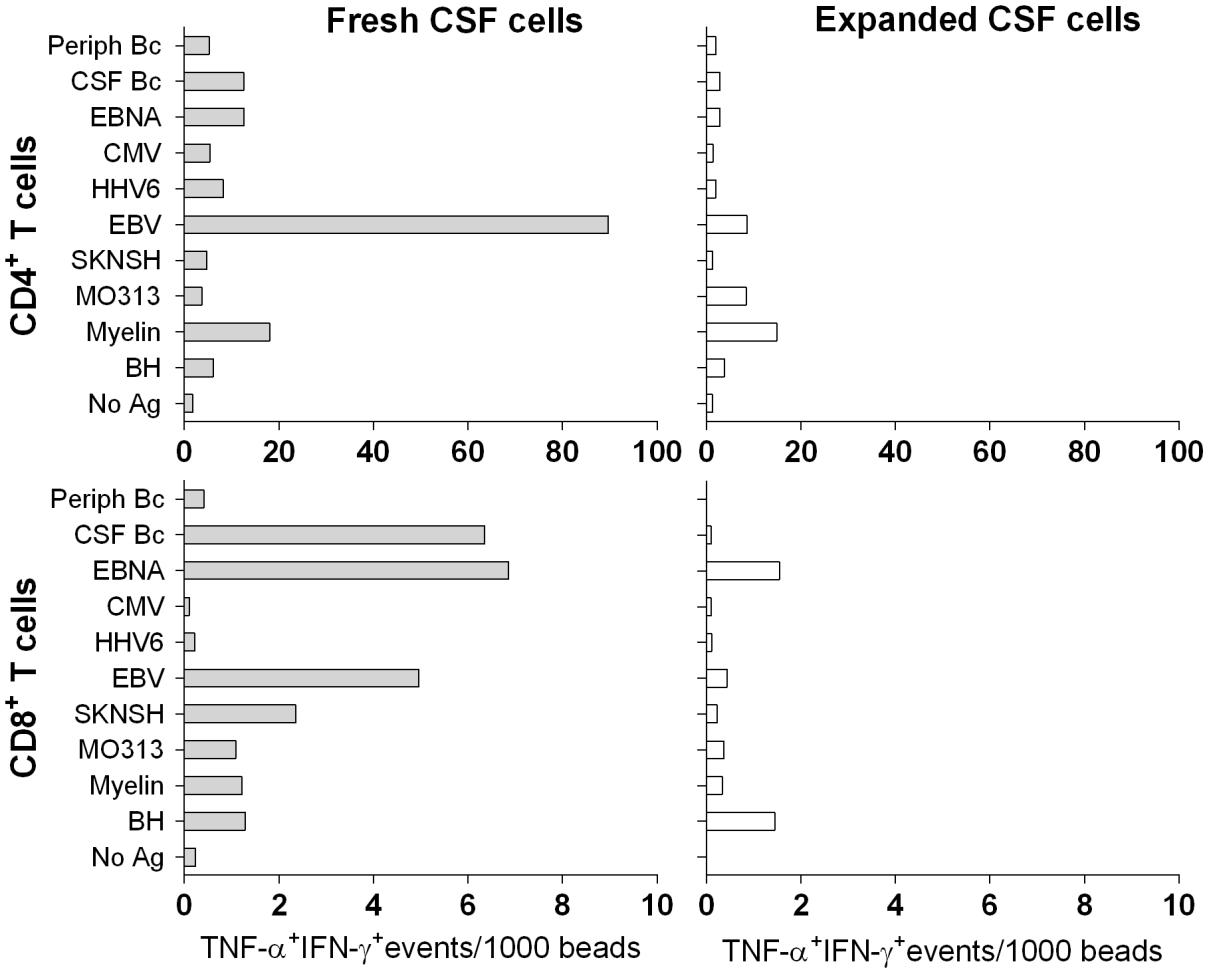
Reduced Ag-specific responses of polyclonally expanded as compared to fresh intrathecal T cells. T cell reactivities of fresh (filled bars) and polyclonally expanded CSF T cells (open bars) from one representative research subject (n = 3) were analyzed using co-cultures with autologous Ag-loaded DCs. TNF-α^+^ IFN-γ^+^ T cell events were standardized to 1000 beads and are depicted for CD4^+^ (upper panels) and CD8^+^ T cells (lower panels).

Given previous estimates of peripheral auto-Ag-specific CD4^+^ T cell precursor frequencies of approximately one per million [24], we aimed to seed a minimum of 1×10^5^ T cells per Ag. The limited numbers of intrathecal T cells required polyclonal expansion to satisfy this goal. Despite successful T cell expansion from 100% of CSF samples averaging 7.98×10^6^ cells, we observed low and poorly reproducible proliferation in response to all tested Ag's (i.e. stimulation indices generally <3). We proceeded to take advantage of the rare opportunity to compare polyclonally expanded and fresh CSF T cells from the same subjects. Although we seeded limited numbers of fresh CSF T cells per condition (3–5×10^3^), these cells led to robust proliferation as compared to polyclonally expanded CSF cells (**Figure 2**). Therefore, we disregarded polyclonally expanded T cells and re-initiated the study from fresh, un-manipulated CSF T cells. To this end, we began cultures of autologous DCs always eight days prior to the LP. Based on pilot experiments (**Figure 2** **and data not shown**), we chose to seed 3×10^3^ T cells cultured in a 1∶1 ratio with Ag-loaded DCs to test all selected Ag's.

### Low T cell reactivities in response to complex auto-Ag's

**Figure 3:**
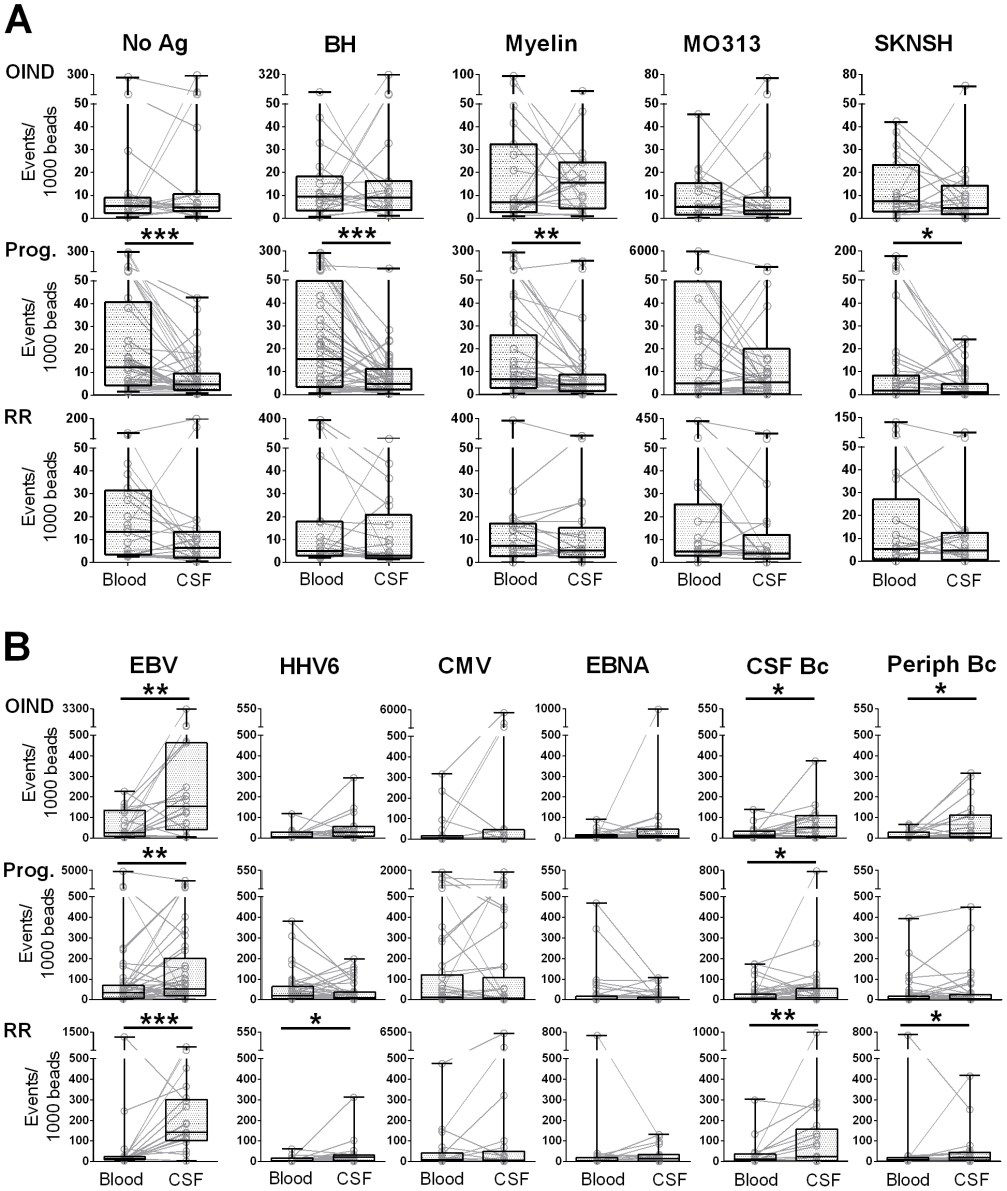
Enrichment of intrathecal T cell reactivities to foreign Ag's. Intracellular cytokine secretion of each research subject was analyzed for IFN-γ^+^, TNF-α^+^ and double positive CD4^+^ T cell events. The sums of all cytokine positive events were normalized to beads. Paired T cell reactivities to unloaded DCs (No Ag) and auto-Ag's (**A**) and foreign Ag's (**B**) are shown for the peripheral (Blood) and intrathecal (CSF) compartment for each subject. Overlaid box plots represent median values with 25^th^ and 75^th^ percentiles; black lines indicate minimum and maximum values; *0.01<p<0.05, **0.001<p<0.01, ***p<0.001.

In general, we observed low CD4^+^ T cell reactivities to auto-Ag's in all three patient groups (OIND, progressive and RRMS patients). As evident in **Figure 3A**, within each cohort we could identify few subjects with high peripheral or CSF T cell reactivities to varied auto-Ag's. However, on a group level, none of the auto-Ag's induced T cell proliferation above the background as defined by the No Ag condition, in which autologous DCs lack exogenously loaded Ag's.

While ranges of peripheral auto-Ag-specific T cell reactivities in OIND patients were slightly higher than those of unloaded DCs, we observed increased levels of “spontaneous” peripheral T cell proliferation in the No Ag condition in both MS cohorts (**Figure 3A**). Although we used identical autologous DCs and serum-free media to establish both peripheral and intrathecal T cell co-cultures, CSF T cells did not proliferate “spontaneously” to unloaded DCs. This difference was most pronounced in progressive MS patients, where it reached statistical significance.

CD8^+^ T cells exhibited even lower T cell responses to auto-Ag's with consistently lower reactivities in CSF than blood (**Figure S1**). These differences reached statistical significance for the No Ag condition, BH and MO313 in both MS cohorts, and myelin and SKNSH for progressive MS patients only.

### Enrichment of intrathecal T cell responses to foreign Ag's

In contrast to auto-Ag's, several foreign Ag's induced CD4^+^ T cell proliferation significantly above the No Ag condition (**Figure 3B**). When comparing reactivities in the blood versus CSF, all diagnostic cohorts had a significant enrichment of intrathecal EBV-specific CD4^+^ T cell responses. The difference was most pronounced in the RRMS cohort, in which we also observed a significant enrichment of HHV6-specific responses, albeit to a lesser extent. However, CSF T cell proliferation to HHV6 seemed to be actually diminished in the progressive MS cohort in comparison to blood. In striking contrast, peripheral and intrathecal CMV reactivities were virtually identical on individual levels in all three cohorts; patients with high peripheral CMV reactivity tended to have a high intrathecal CMV reactivity and vice versa. Finally, we observed slight increases in CSF reactivities to EBV-transformed B cell lysates (CSF- and blood-derived B cells) throughout all diagnostic cohorts to varying degrees (**Figure 3B**), with the RRMS cohort having the greatest enrichment.

We performed the same analyses for CD8^+^ T cells. Overall, the intrathecal enrichment described for CD4^+^ T cells was not detectable (**Figure S1**). Marginally increased intrathecal EBV-specific T cell reactivities were apparent in the OIND and RRMS cohorts. Progressive patients, on the other hand, had significantly lower HHV6-specific T cell responses in the CSF than in the blood, supporting the analogous change seen in CD4^+^ T cells (**Figure S1 and** **Figure 3B**).

### Phenotype of Ag-specific intrathecal and peripheral T cells

Utilizing ICCS, we assessed the secretion of IL-2, IL-17, IFN-γ, TNF-α, GM-CSF and BDNF. In all diagnostic groups we did not observe a significant secretion of IL-2, IL-17, GM-CSF and BDNF in response to any of the tested Ag's higher than the No Ag condition (**data not shown**). As almost all Ag-specific cells expressed TNF-α and IFN-γ we considered the possibility that the conditions of the ICCS assay may favor their detection as compared to the detection of other cytokines, which may be produced faster (e.g. IL-2 and GM-CSF) or slower (e.g. BDNF) than TNF-α and IFN-γ.

Therefore, we next collected supernatants from replicate co-cultures of most robustly proliferating CSF T cells and measured cytokine secretion using multiplex membrane antibody arrays. Again, this methodology did not demonstrate any significant accumulation of IL-17 or GM-CSF (**Figure S2**). However, this was also true for IFN-γ, which we detected by ICCS. We concluded that analytes detected in culture supernatants represent the difference between production and consumption. Confirmation that the assay worked properly came from clear accumulation of TNF-α and chemokines, such as IL-8, IP-10 and MCP-1 in Ag-specific conditions (**Figure S2**).

**Figure 4:**
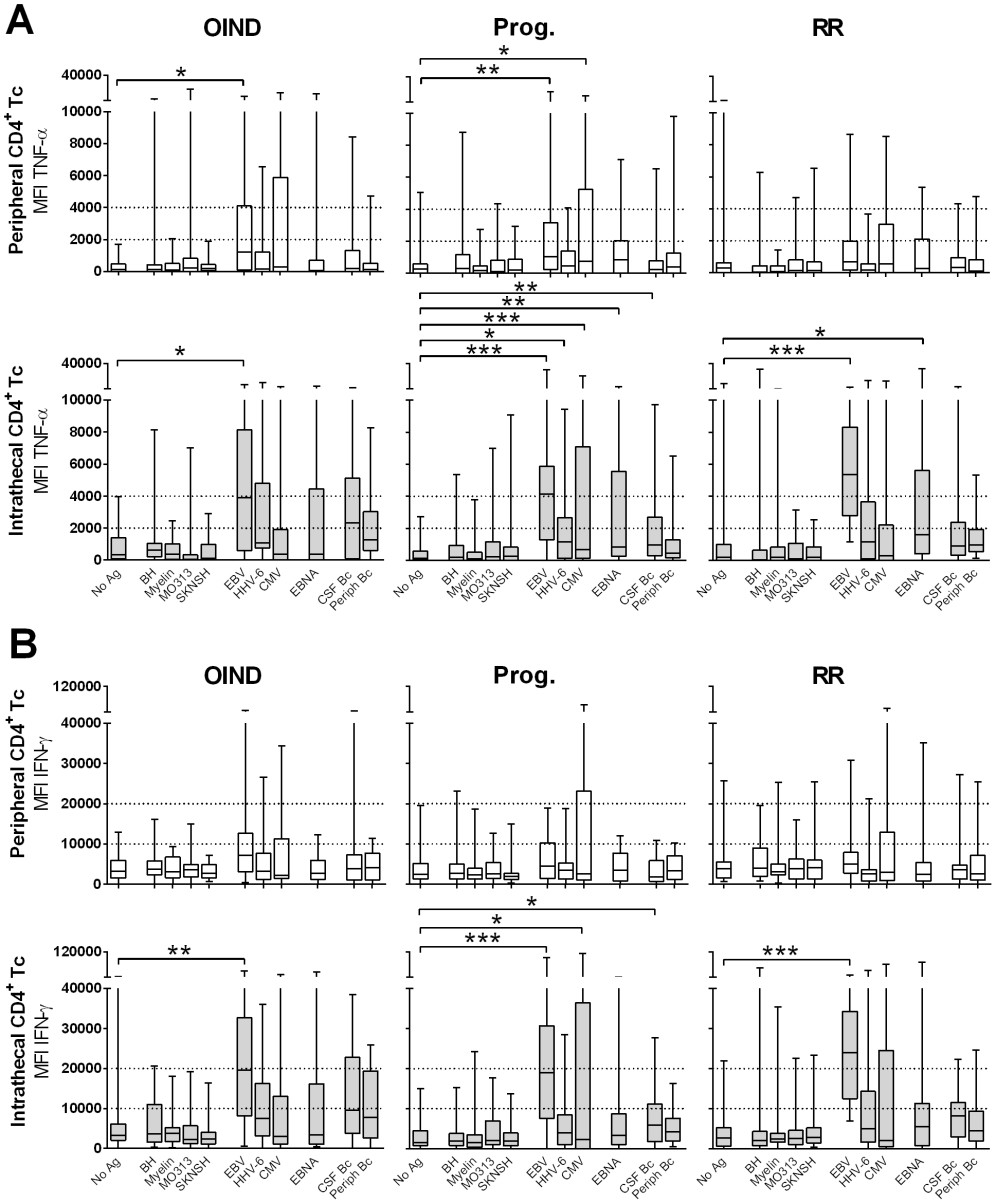
MFIs of peripheral and intrathecal CD4^+^ T cells. MFIs of TNF-α- (**A**) and IFN-γ-producing (**B**) peripheral (upper panels) and intrathecal (lower panels) CD4^+^ T cells are shown for OIND, progressive (Prog.) and relapsing-remitting (RR) patients in response to all candidate Ag's. *0.01<p<0.05, **0.001<p<0.01, ***p<0.001.

Consequently, we focused our phenotypical analysis on the two Th1 cytokines that were consistently expressed in the vast majority of Ag-specific T cell cultures: TNF-α and IFN-γ. Comparison of the MFIs of these cytokines, gated on all CD4^+^ (**Figures 4A, B**) or CD8^+^ T cells (**Figure S3**) provided information complementary to the one presented previously, as it captures the group level of cytokines secreted in response to each Ag. In peripheral blood, only EBV- and CMV-specific CD4^+^ T cells expressed significantly higher TNF-α (**Figure 4A** **, upper panel**) as compared to the No Ag condition. However, this was only observed in OIND and progressive MS patients.

In contrast, robust differences were observed in CSF T cells, where EBV-specific T cells secreted significantly higher quantities of both cytokines, in comparison to the control condition in all patient cohorts. The absolute increase in IFN-γ and TNF-α MFIs of EBV-specific T cells between blood and CSF was the highest in the RRMS cohort, consistent with the observations previously noted from proliferation assays. Progressive patients had a significantly elevated production of TNF-α (**Figure 4A** **, lower panel**) and IFN-γ (**Figure 4B** **, lower panel**) to several additional Ag's, including HHV6, CMV, EBNA and EBV-transformed CSF B cell lysate (CSF Bc). This was surprising when we recollect that this cohort has diminished intrathecal CD4^+^ and CD8^+^ T cell proliferation to HHV6. RRMS patients had also remarkably increased TNF-α production to EBNA.

Analogous changes were observed in CD8^+^ T cells especially for TNF-α production, which was again significantly increased in progressive MS patients to several Ag's including EBV, CMV and EBNA (**Figure S3**).

### Differential phenotypes of peripheral and intrathecal T cells

**Figure 5:**
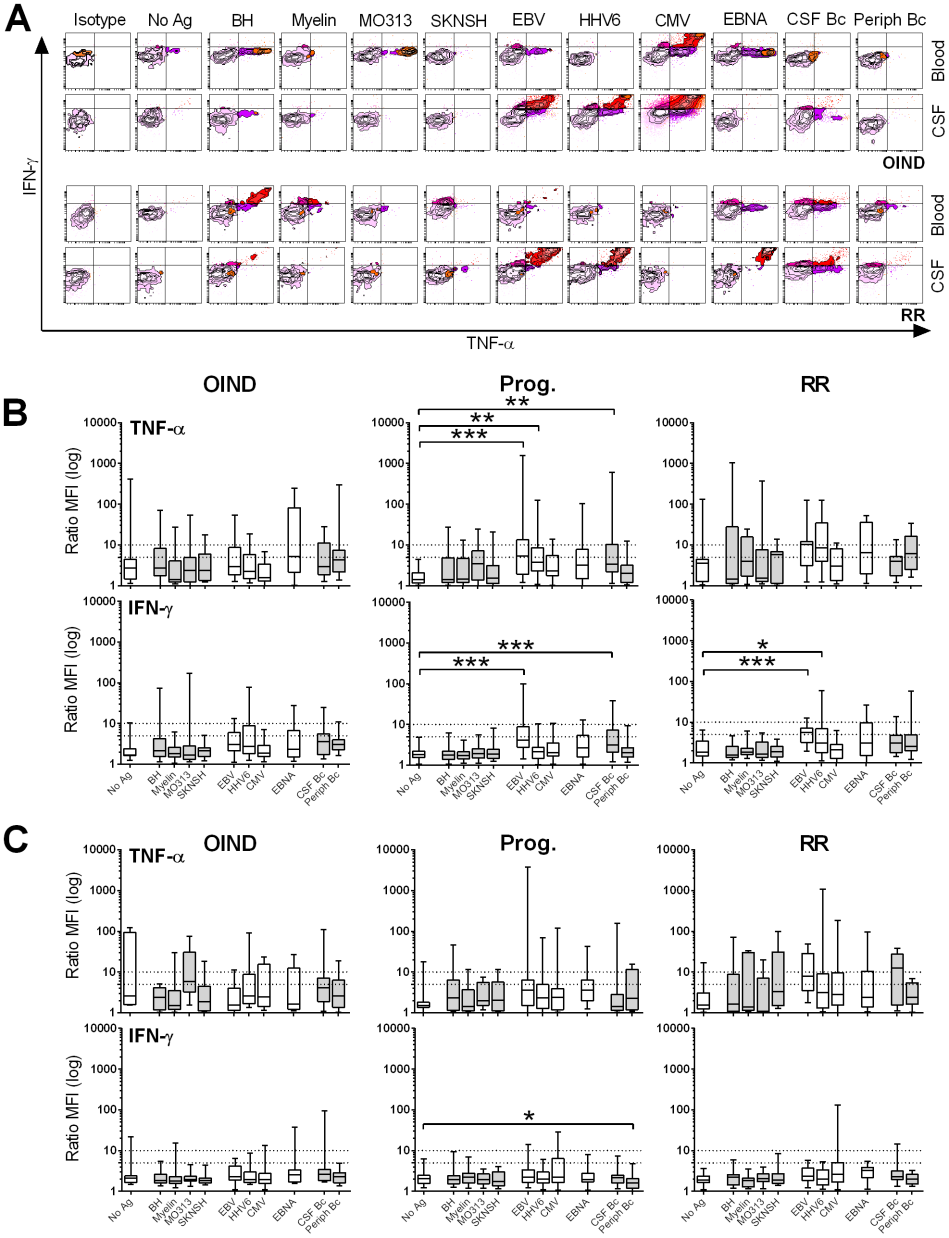
Differential phenotypes of peripheral and intrathecal CD4^+^ T cells in response to foreign Ag's. (**A**) FACS plots illustrate intracellular TNF-α and IFN-γ secretion by peripheral (Blood) and intrathecal (CSF) CD4^+^ T cells in response to all candidate Ag's. Gating is based on the isotype controls (far left plots). Upper panels correspond to one representative OIND patient; lower panels correspond to one representative un-treated RRMS patient. MFI ratios of intrathecal to peripheral CD4^+^ (**B**) and CD8^+^ T cells (**C**) were calculated for TNF-α (upper panel) and IFN-γ (lower panel), and are shown for OIND, progressive and RRMS patients. MFI ratios are depicted as log-transformed data. *0.01<p<0.05, **0.001<p<0.01, ***p<0.001.

Careful analysis of the data described in **Figure 3** suggest a phenotypical difference between peripheral and intrathecal Ag-specific T cells beyond increased EBV-specific T cell reactivities. Indeed, individual FACS plots demonstrated higher cytokine production by CSF T cells, even when patients had EBV-specific T cells in both compartments (**Figure 5A**). To formally test this hypothesis, we calculated the ratios between CSF and blood of TNF-α and IFN-γ MFIs for all conditions in CD4^+^ and CD8^+^ T cells. Virtually all intrathecal T cells produced higher levels of both TNF-α and IFN-γ independent of the particular Ag (i.e. MFI ratios >1; **Figures 5B, C**).

Notably, EBV-specific CD4^+^ T cell responses elicited significantly greater quantities of IFN-γ in comparison to the No Ag condition for progressive and RRMS patients. Similar intrathecal enrichment was visible in RRMS patients for HHV6 and in progressive patients for CSF Bc. TNF-α MFIs reached statistical significance for EBV, HHV6 and CSF Bc in the progressive MS cohort only (**Figure 5B**).

### Daclizumab-induced changes of peripheral and intrathecal T cell reactivities

While unbiased, properly controlled observational studies may disclose disease-specific changes, they cannot determine whether or not these changes are pathophysiologically relevant. Only application of therapies that successfully inhibit disease activity can identify those biological changes that may play a pathogenic role in the disease process. We applied this rationale to our study by comparing un-treated to long-term DAC HYP-treated RRMS patients. DAC HYP is a monoclonal antibody directed against CD25 that effectively inhibits MS disease activity based on clinical and MRI criteria [25]–[27].

**Figure 6:**
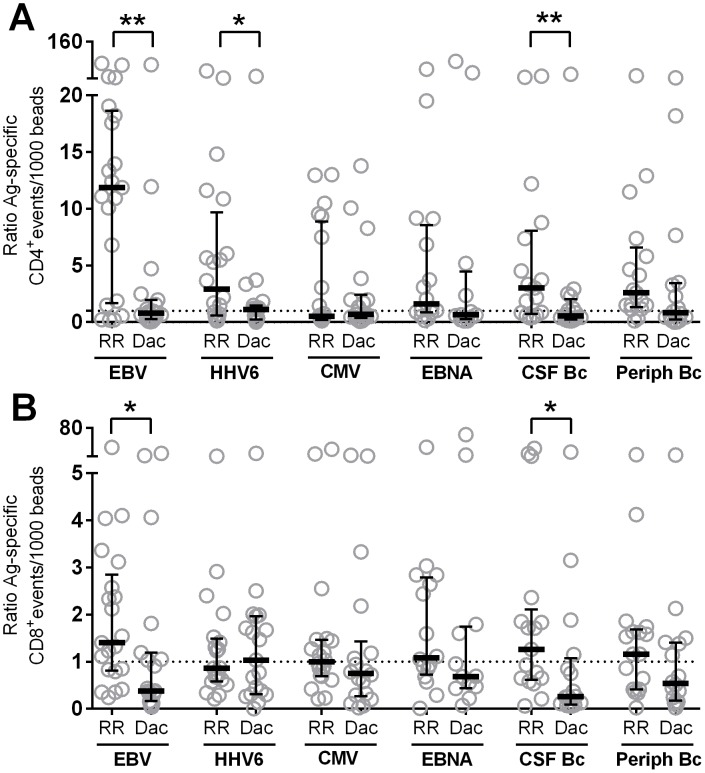
Daclizumab-induced normalization of Ag-specific T cell responses. Intracellular cytokine secretion of each research subject was analyzed for IFN-γ^+^, TNF-α^+^ and double positive T cell events. The sums of all cytokine positive events were normalized to beads. Ratios of intrathecal to peripheral T cell reactivities of un-treated (RR) and DAC HYP-treated RRMS patients (Dac) were calculated to foreign Ag's for CD4^+^ (**A**) and CD8^+^ T cells (**B**). Ratios greater than one (dotted line) indicate enrichment of Ag-specific T cell events in the intrathecal compartment; ratios lower than one represent less pronounced intrathecal T cell responses. Horizontal bars represent median values; vertical lines represent interquartile ranges. *0.01<p<0.05, **0.001<p<0.01, ***p<0.001.

In order to capture differences between T cell reactivities in both compartments, we calculated ratios from intrathecal and peripheral T cell responses to various Ag's. Reactivity-based ratios did not reveal any considerable differences between CD4^+^ and CD8^+^ T cells (**Figure S4**) in response to auto-Ag's, with generally higher reactivities found in the periphery as compared to CSF (i.e. ratios <1). However, DAC HYP profoundly reduced the ratios in response to EBV and CSF Bc in both CD4^+^ and CD8^+^ T cells, and HHV6 in CD4^+^ T cells alone (**Figures 6A, B**). These changes were not due to immunosuppressive effects of DAC HYP, but instead coincided with a decreased reactivity in the CSF and increased reactivity in the blood, especially apparent for CD4^+^ T cell responses to EBV (**Figure S4**).

## Discussion

Despite ample efforts to unravel the cause(s) of MS, the antigenic target(s) have not been determined yet. In our current study, we employed co-cultures of freshly obtained CSF T cells with autologous mDCs pre-loaded with complex candidate Ag's. Utilization of this DC-based system provided several important advantages over published studies: First, it allowed physiological processing of Ag's (including their post-translational modifications) and selection of immunodominant epitopes for each HLA-allele in a patient-specific assembly of MHC-I and MHC-II molecules. Second, it assured that the observed differences between the diagnostic categories reside in the T cell compartment, as we routinely checked the phenotype of *in vitro* generated autologous mDCs to guarantee comparability for each subject. T cells cannot serve as APCs in this assay, because they lack the proteolytic machinery for endogenous processing of complex Ag's. If Ag lysates contained some short peptides that could be exogenously loaded onto MHC molecules, we have previously reported that CSF T cells derived from MS and OIND cohorts have comparable expression of MHC molecules [15]. Lastly, we were able to study CD4^+^ and CD8^+^ T cell reactivities in parallel. Evidence for the functionality of our assay originates from the observed coordinated behavior of CD4^+^ and CD8^+^ T cells specific for identical Ag's. While EBV-specific CD4^+^ and CD8^+^ T cells for example were generally expanded in the CSF as compared to blood, patients with CMV reactivities had either high CD4^+^ and CD8^+^ T cell levels in both compartments or no reactivity at all. Indeed, physiological immune responses to complex Ag's, such as viruses should comprise synchronized CD4^+^ and CD8^+^ T cells responses.

After meticulous selection of the candidate auto-Ag's we observed disappointingly low T cell reactivities throughout all patient cohorts. Although we could clearly identify individual patients with strong reactivities to some of the tested auto-Ag's, these individuals were equally distributed among all diagnostic cohorts. These results contradict some (but not all) previously published studies, which suggested an enrichment of auto-reactive T cells in the blood and/or CSF of MS patients. Yet, those studies rarely used OIND controls. We will not repeat the technical drawbacks of previous studies listed in the introduction and which served as an impetus for our current study. Instead, we will focus on studies that question the validity of belief that myelin represents the major antigenic target in MS. For instance, T cells specific for myelin epitopes have been observed in similar frequencies in children with MS, non-autoimmune CNS insult and type 1 diabetes (T1D), whereas T cells specific for pancreatic auto-Ag's have been identified in the T1D cohort only [28]. Furthermore, no correlation was found between the precursor frequency or activation status of myelin-specific CD4^+^ T cells and MS disease activity [29]. Analyses of serum and CSF revealed that humoral responses in MS patients do not include high affinity auto-Ab's to myelin Ag's, such as myelin oligodendrocyte glycoprotein (MOG) [30], [31]. Antibody data are informative particularly as B cells need help from CD4^+^ T cells that are specific for identical auto-Ag's to initiate affinity maturation and isotype switching. These processes are necessary for the secretion of high-affinity immunoglobulin G Ab's by plasma cells. Thus, the lack of high affinity Ab's against myelin Ag's in MS is in striking contrast to T1D, where high-affinity auto-Ab's to the target tissue are present even before the initiation of T-cell mediated destruction of pancreatic islets and persist lifelong [32], [33]. In contrast to MS, high-affinity Ab's against MOG epitopes are in fact relatively frequent in acute demyelinating encephalomyelitis [31]. Therefore, complementary pieces of evidence do not support a central role of common myelin Ag's in the pathogenesis of MS.

Yet, we believe that our study does not represent a definite evidence that Ag's which are less prevalent in the CNS (such as the potassium channel KIR4.1 [34] or the axoglial contactin-2/TAG-1 [35]) may not be the target of the pathogenic immune responses in MS. The precursor frequency of *peripheral* T cells specific for single epitopes derived from *intrathecal* Ag's, is estimated to be one per million T cells. However, under unique conditions where a single epitope drove intrathecal inflammation (i.e. in an altered peptide ligand trial in MS), the precursor frequency of MBP_83-99_-specific CD4^+^ T cells was equal to or higher than 1/1000 in the blood and 1/100 in the CSF [36]. Because our assay is capable of detecting such highly prevalent T cell responses, we conclude that if auto-Ag-specific T cells drive disease processes in MS their precursor frequency in the CSF has to be below 1/1000. Also, by using complex Ag's instead of peptides, we tested hundreds of naturally processed epitopes within each condition. With the epitope spreading theory, one would expect an increase in the number of pathogenic T cell clonotypes with disease duration, but this was not observed in our current study. Therefore, we conclude that it is unlikely that more than 1/1000 T cells in the CSF of MS patients irrespective of the disease duration target an intrathecal Ag. Consequently, it may be impossible with current technologies to reliably detect infrequent auto-Ag-specific T cell responses in the CSF of MS patients, unless one focuses on single Ag's utilizing all CSF T cells for such an experiment. Unfortunately, we believe that any type of *in vitro* pre-expansion does not present an adequate solution, as terminally differentiated T cells have a poor proliferative capacity in contrast to their efficient cytokine secretion [37], and this is precisely the T cell phenotype we identified in the intrathecal compartment of progressive MS patients.

In contrast to CNS Ag's, we found thought-provoking differences between the diagnostic cohorts in response to viral Ag's. CMV served as an excellent control, as we observed no preferential recruitment or retention of CMV-specific T cells in the intrathecal compartment. Unexpectedly, strong intrathecal enrichment occurred in response to EBV and EBV-related Ag's. Although present in all patient cohorts, this enrichment was most pronounced in MS patients, who also had the highest intrathecal production of the pro-inflammatory cytokines IFN-γ and TNF-α. Peripheral T cell reactivities in particular were diminished whereas CSF T cell responses were amplified in RRMS compared to OIND patients. The coinciding reductions of peripheral EBV-specific T cell reactivities in RRMS patients strongly suggest their preferential recruitment into the CNS rather than intrathecal expansion alone. Strikingly, long-term treatment of RRMS patients with DAC HYP increased peripheral and decreased intrathecal T cell reactivities, thus normalizing the enrichment of EBV-specific T cell responses. This is intriguing given DAC HYP's association with strong inhibition of formation of MS lesions and stabilization of clinical scores [25], [38].

We consider the uniform intrathecal enrichment of EBV-specific T cells across all neuro-inflammatory groups adequate evidence that EBV is not the primary target of the pathogenic immune responses in all of these varied conditions. This interpretation is supported by published studies that demonstrated comparable or even higher levels of intrathecal Ab's against EBV proteins in OIND subjects, as compared to MS [39]–[41]. Because EBV infection prompts phenotypic B cell maturation [42], EBV-infected B cells are likely recruited to the inflamed tissue along with non-specific inflammatory cell infiltrates. It has been argued that the CNS provides a uniquely favorable environment to B cells due to the secretion of B cell growth factors like BAFF [43]. The presence of EBV Ag's in the CNS [43] and the possibility for latent EBV reactivation within the inflammatory focus remains highly debated [43], but we consider it likely. EBV reactivations have been observed in peripheral blood of MS patients (where it may coincide with increased MRI activity) and reactivations occur periodically with all herpes viruses [43]. Such reactivation would induce a secondary expansion and retention of EBV-specific T cells in the CNS tissue to suppress the development of active infectious processes. Our data indicate that EBV-specific T cells are not retained in the CNS regardless of the presence of EBV, because CMV-specific T cells did not exhibit a similar behavior. Thus, indirectly, our observation of intrathecal EBV-specific T cell enrichment in inflammatory CNS conditions provides support to those studies that identified intrathecal expression of EBV Ag's. EBV reactivation in the intrathecal compartment is probably as infrequent as reactivation of neurotropic herpes viruses [44] and therefore would be expected to be undetectable by current technologies [45], unless it leads to virus-mediated immunopathology.

Nevertheless, CSF enrichment of EBV-specific T cells is not exclusive to MS, but rather represents a characteristic feature of any intrathecal inflammation. This does not invalidate a possible role of EBV in the initiation or exacerbation of the MS disease process. In fact, epidemiological associations between EBV and immune-mediated diseases, such as MS but also systemic autoimmune diseases [43] suggest that EBV may play an important role in sustaining chronic inflammation, even if it does not represent the primary target of the immune response. EBV reactivation and related EBV-specific T cell responses may lead to activation of local APCs [46] and secretion of pro-inflammatory cytokines that may mediate damage to the surrounding CNS tissue [43].

HHV6 has likewise been linked to MS in several studies [43]. We detected increased intrathecal CD4^+^ T cell proliferation in response to HHV6 in RRMS and cytokine secretion in progressive patients. But our data also show enhanced intrathecal CD4^+^ T cell proliferation to HHV6 in the OIND cohort. Therefore, as with EBV, increased reactivity to HHV6 does not appear to be specific for MS, but is rather driven by intrathecal inflammation, which is the conclusion derived from intrathecal Ab studies against HHV6 as well [47], [48]. HHV6 can reside in the CNS either as neurotropic variant [49] or can gain access to this compartment via infected lymphocytes and monocytes [43]. Latent herpes viruses reactivate during episodes of stress and intrathecal inflammation may provoke more frequent reactivation of HHV6. Although our study lacks direct evidence, it is plausible that analogous to EBV, this cyclic Ag-driven expansion of HHV6-specific T cells in the CNS tissue contributes to the disease activity.

It is interesting to consider these explanations (of EBV and HHV6 infections being involved in the disease process) in view of our data obtained from patients on long-term DAC HYP therapy who experienced stabilization of their disease processes. In this cohort, we observed a striking reversal of intrathecally enriched EBV- and HHV6-specific T cell reactivities. We have recently reported that DAC HYP inhibits differentiation of innate lymphoid cells into lymphoid tissue inducer cells, which may be important for the formation or maintenance of lymphoid aggregates sustaining chronic inflammatory responses [50]. In this context, our current data could be interpreted as indirect evidence for the inhibition of meningeal inflammation leading to egress of previously recruited B cells (including those that might have been EBV-infected) and T cells from the intrathecal compartment back to the periphery. Thus, the DAC HYP-induced reversal of enriched intrathecal EBV- and HHV6-specific T cell reactivities supports the hypothesis that T cell responses to these viruses might contribute to the disease activity of MS.

Finally, HHV6-specific T cell responses brought to our attention an inverse relationship between proliferation and cytokine secretion in the progressive MS cohort. Terminal differentiation of Ag-specific T cells likely accounts for this observation [37]. Repeated rounds of stimulation render T cells extremely efficient cytokine producers (especially IFN-γ and TNF-α, but not IL-2, which is no longer secreted at this point [51]), earning the designation of terminal effector T cells with a strong cytotoxic potential, but limited proliferative capacity [37]. Although these phenotypical differences may seem rather subtle to be biologically meaningful, systems biology indicates otherwise: small differences, such as a higher production of greater numbers of pro-inflammatory cytokines determine the expression of the disease, as organisms integrate such inputs in a non-linear manner. For example, while injection of either IFN-γ or TNF-α to the subarachnoid space of MOG-immunized Dark Agouti rats led to small areas of subpial cortical demyelination, the combination of both cytokines exhibited a strong synergistic effect [43].

Thus, our data support the model which involves the recruitment of Ag-specific T cells from the periphery to the CSF during active inflammation, as seen in RRMS and OIND patients. Subsequent to the clearance of active inflammation, the majority of the recruited T cells dies or returns to the periphery, while few cells are retained in the CNS tissue for immunosurveillance. In chronic inflammation, Ag-specific T cell activation can occur entirely within the CNS compartment upon the establishment of tertiary lymphoid tissue. The presence of intrathecal Ag's stimulates repetitive T cell activation followed by terminal differentiation. Rather than continuing expansion, these cells sustain robust inflammatory responses via highly efficient cytokine production. The inefficacies of immunomodulatory therapies at this stage of MS can be attributed to several phenomena. Compartmentalization denies the access of systemically administered therapies to the intrathecal compartment (which is especially true for large molecules) and terminal differentiation limits the efficacy of therapies preferentially targeting cells in the proliferation cycle (i.e. immunosuppressive small molecular agents). Therefore, intrathecally applicable therapies targeting immune cells outside of the proliferation cycle are likely required to treat progressive MS patients with fully compartmentalized immune responses.

## Supporting Information

Figure S1 **Peripheral and intrathecal CD8^+^ T cell reactivities to auto- and foreign Ag's.** Intracellular cytokine secretion of each research subject was analyzed for IFN-γ^+^, TNF-α^+^ and double positive CD8^+^ T cell events. The sums of all cytokine positive events were normalized to beads. Paired T cell reactivities to unloaded DCs (No Ag) and auto-Ag's (**A**) and foreign Ag's (**B**) are shown for the peripheral (Blood) and intrathecal (CSF) compartment for each subject. Overlaid box plots represent median values with 25^th^ and 75^th^ percentiles; black lines indicate minimum and maximum values; *0.01<p<0.05, **0.001<p<0.01, ***p<0.001.
(TIF)

Figure S2 **Negligible IL-17 and GM-CSF levels in cell culture supernatants.** CSF T cell superantants were collected seven days after establishment of fresh co-cultures and analyzed for pro-inflammatory cytokines and chemokines. The groups consisted of supernatants from either unloaded (No Ag; n = 30), EBV-, HHV6- or CMV-loaded co-cultures (n = 31). Auto-Ag analysis involved 34 supernatants in total obtained from a combination of BH, Myelin, MO313 and SKNSH co-cultures. Results for TNF-α, IFN-γ, IL-17, GM-CSF, IL-8, IP-10 and MCP-1 are presented in the bar graph as arbitrary units.
(TIF)

Figure S3 **MFIs of peripheral and intrathecal CD8^+^ T cells.** MFIs of TNF-α- (**A**) and IFN-γ-producing (**B**) peripheral (upper panels) and intrathecal (lower panels) CD8^+^ T cells are shown for OIND, progressive (Prog.) and relapsing-remitting (RR) patients in response to all candidate Ag's. *0.01<p<0.05, **0.001<p<0.01, ***p<0.001.
(TIF)

Figure S4 **Daclizumab-induced changes of peripheral and intrathecal T cell reactivities.** Intracellular cytokine secretion of each research subject was analyzed for IFN-γ^+^, TNF-α^+^ and double positive T cell events. The sums of all cytokine positive events were normalized to beads. Ratios of intrathecal to peripheral auto-Ag-specific T cell reactivities of un-treated (RR) and DAC HYP-treated RRMS patients (Dac) were calculated for CD4^+^ (**A**) and CD8^+^ T cells (**B**). Ratios greater than one (dotted line) indicate enrichment of Ag-specific T cell events in the intrathecal compartment; ratios lower than one represent less pronounced intrathecal T cell responses. Horizontal bars represent median values; vertical lines represent interquartile ranges. (**C**) CD4^+^ T cell reactivities to foreign Ag's are shown for blood (upper panel) and CSF (lower panel) for the same patient cohorts described above. Box plots represent median values with 25^th^ and 75^th^ percentiles; black lines indicate minimum and maximum values. *0.01<p<0.05, **0.001<p<0.01, ***p<0.001.
(TIF)
