# Supplementary figures and images for: A Complex Role of Herpes Viruses in the Disease Process of Multiple Sclerosis
Source: PLoS One. 2014 Aug 22;9(8):e105434. doi: 10.1371/journal.pone.0105434 (PMC4141762; doi:10.1371/journal.pone.0105434)

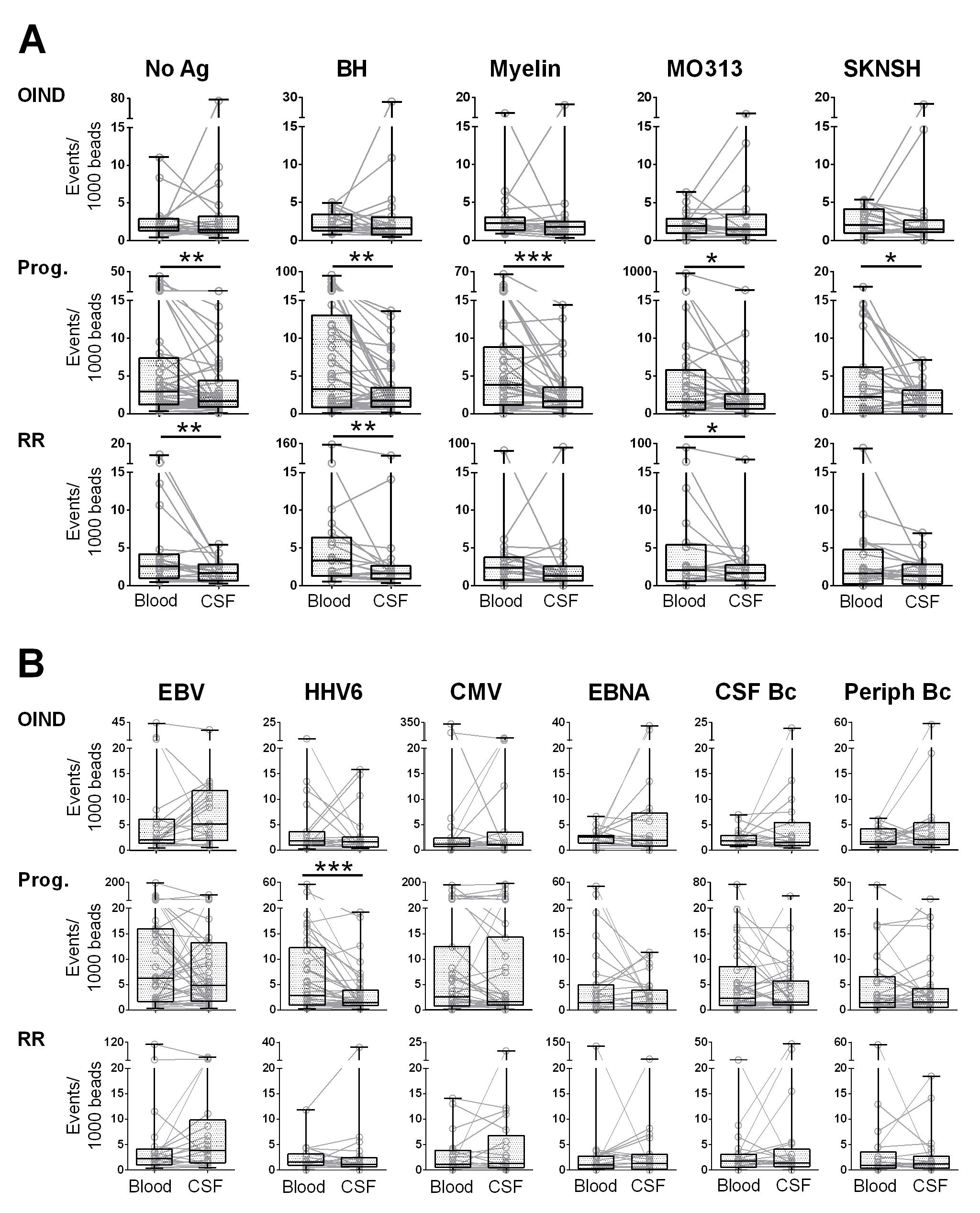

Supplement: Figure S1 — Peripheral and intrathecal CD8+ T cell reactivities to auto- and foreign Ag's. Intracellular cytokine secretion of each research subject was analyzed for IFN-γ+, TNF-α+ and double positive CD8+ T cell events. The sums of all cytokine positive events were normalized to beads. Paired T cell reactivities to unloaded DCs (No Ag) and auto-Ag's (A) and foreign Ag's (B) are shown for the peripheral (Blood) and intrathecal (CSF) compartment for each subject. Overlaid box plots represent median values with 25th and 75th percentiles; black lines indicate minimum and maximum values; *0.01<p<0.05, **0.001<p<0.01, ***p<0.001. (TIF) [file pone.0105434.s001.tif]

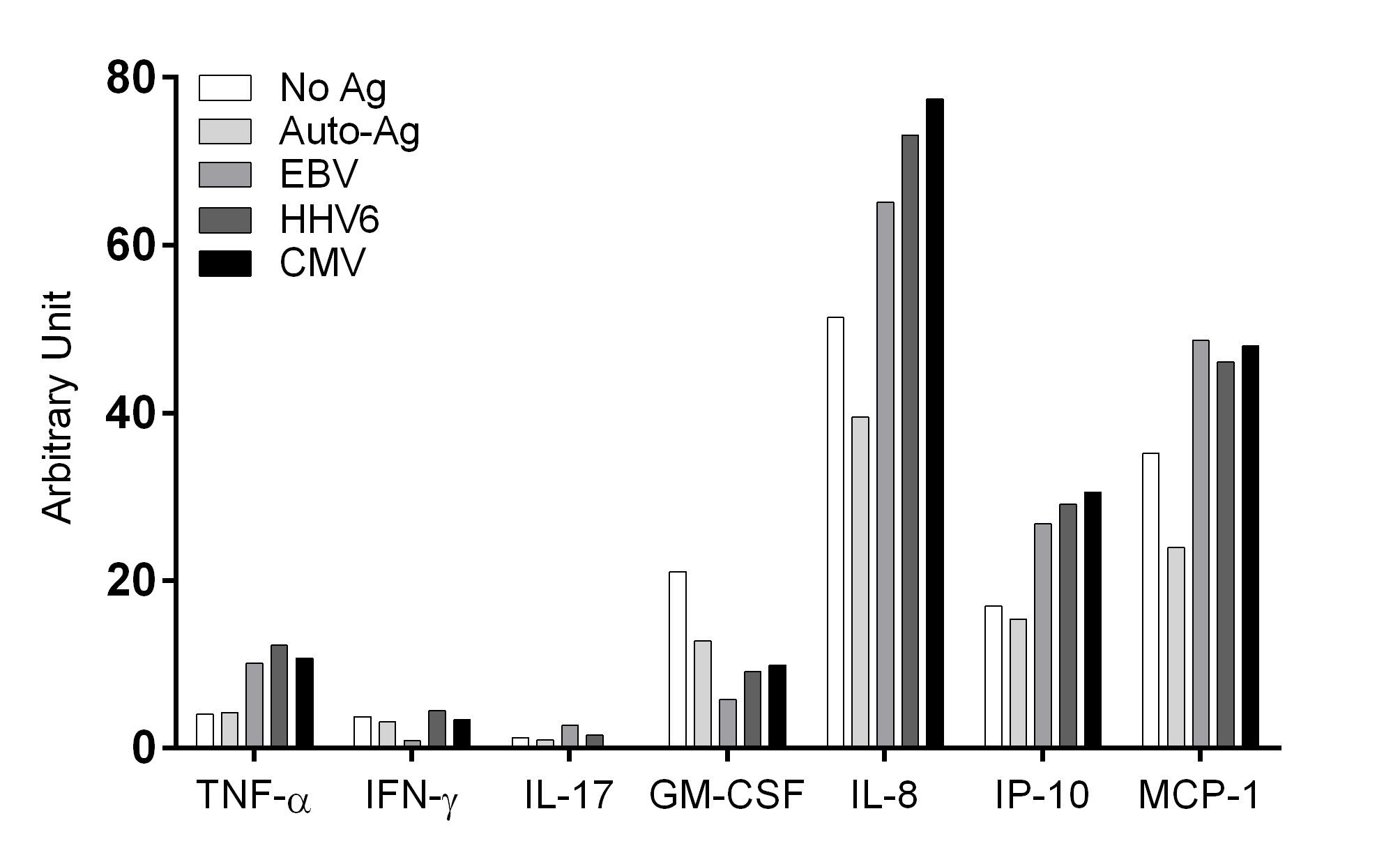

Supplement: Figure S2 — Negligible IL-17 and GM-CSF levels in cell culture supernatants. CSF T cell superantants were collected seven days after establishment of fresh co-cultures and analyzed for pro-inflammatory cytokines and chemokines. The groups consisted of supernatants from either unloaded (No Ag; n = 30), EBV-, HHV6- or CMV-loaded co-cultures (n = 31). Auto-Ag analysis involved 34 supernatants in total obtained from a combination of BH, Myelin, MO313 and SKNSH co-cultures. Results for TNF-α, IFN-γ, IL-17, GM-CSF, IL-8, IP-10 and MCP-1 are presented in the bar graph as arbitrary units. (TIF) [file pone.0105434.s002.tif]

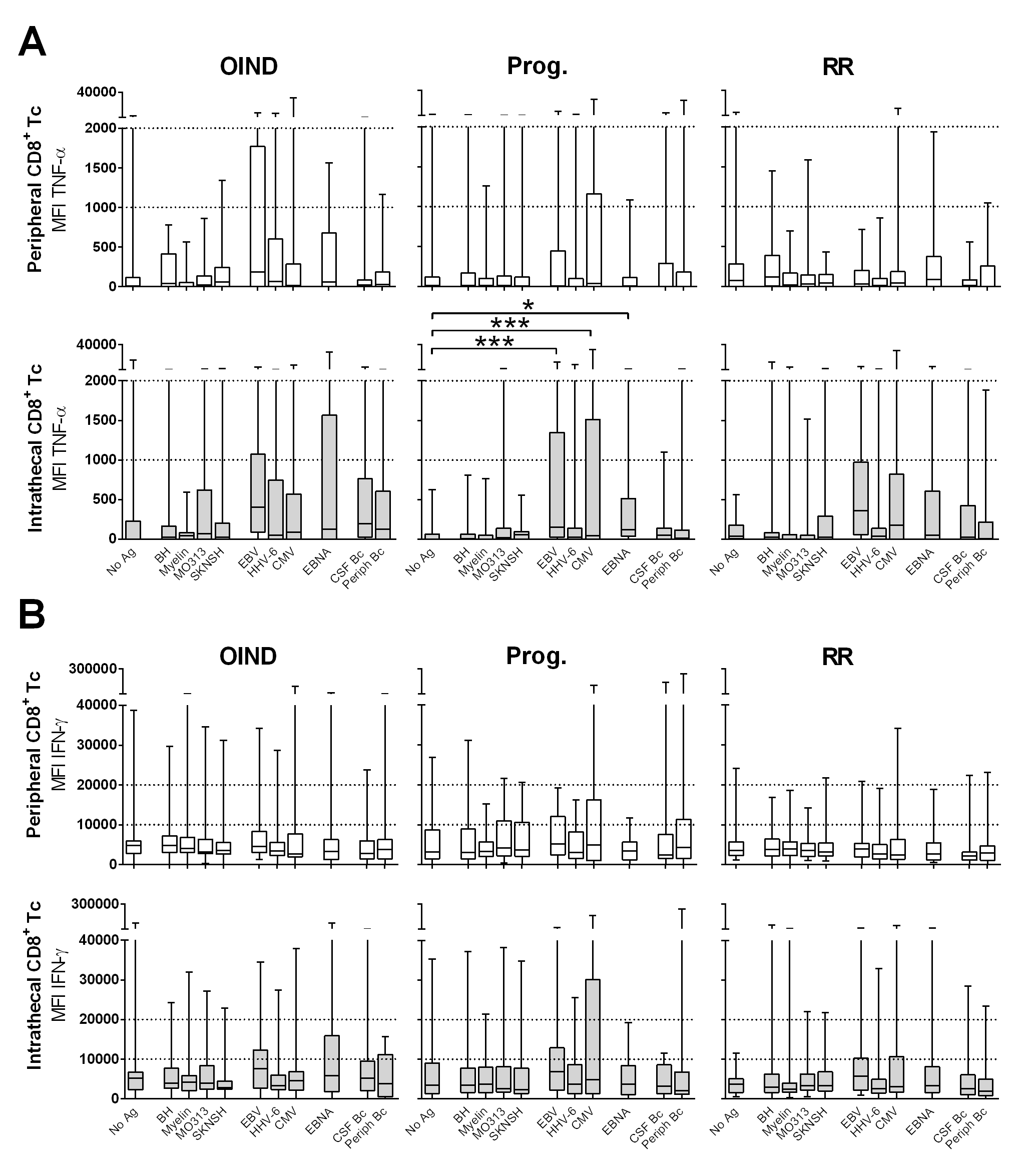

Supplement: Figure S3 — MFIs of peripheral and intrathecal CD8+ T cells. MFIs of TNF-α- (A) and IFN-γ-producing (B) peripheral (upper panels) and intrathecal (lower panels) CD8+ T cells are shown for OIND, progressive (Prog.) and relapsing-remitting (RR) patients in response to all candidate Ag's. *0.01<p<0.05, **0.001<p<0.01, ***p<0.001. (TIF) [file pone.0105434.s003.tif]

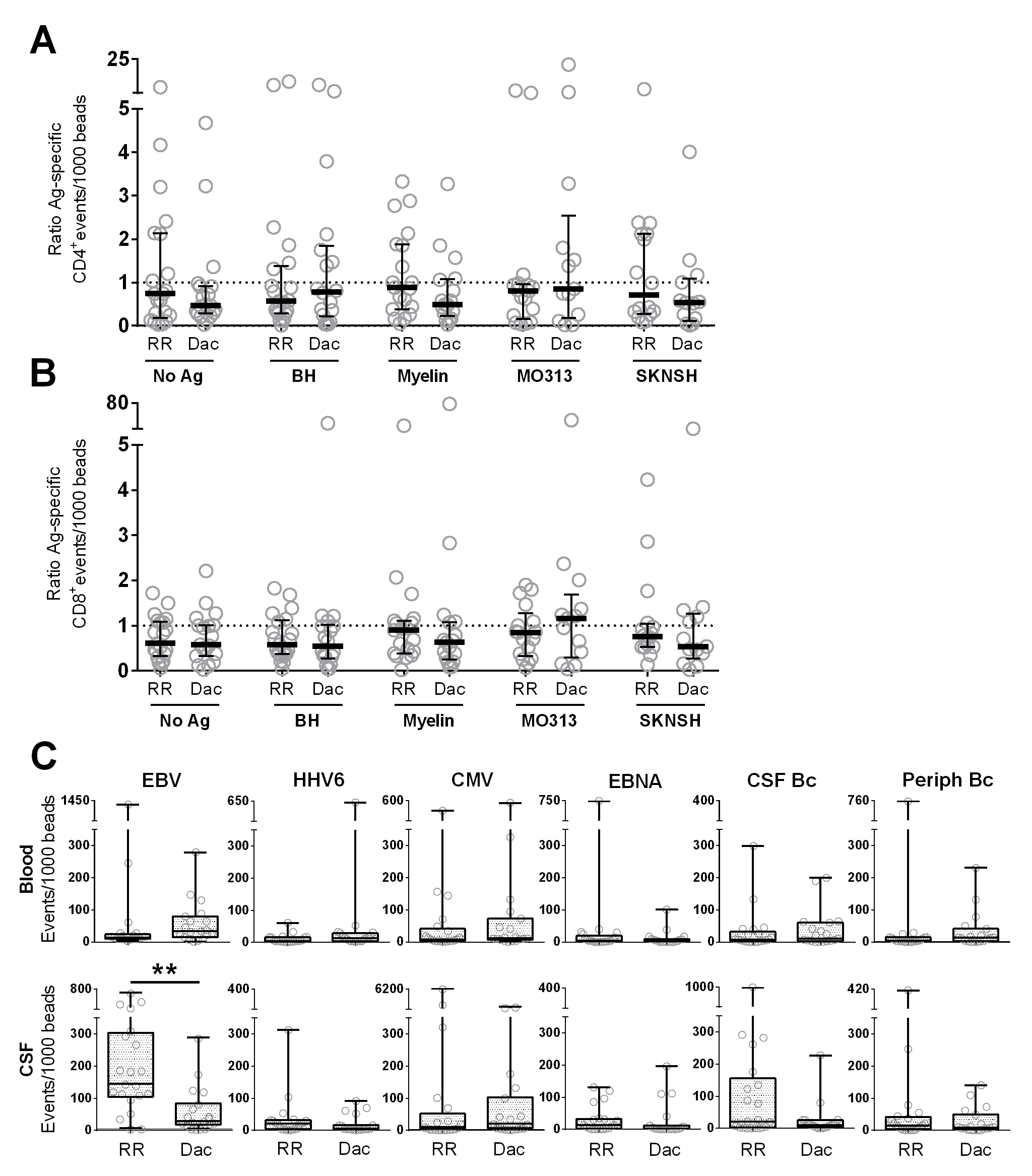

Supplement: Figure S4 — Daclizumab-induced changes of peripheral and intrathecal T cell reactivities. Intracellular cytokine secretion of each research subject was analyzed for IFN-γ+, TNF-α+ and double positive T cell events. The sums of all cytokine positive events were normalized to beads. Ratios of intrathecal to peripheral auto-Ag-specific T cell reactivities of un-treated (RR) and DAC HYP-treated RRMS patients (Dac) were calculated for CD4+ (A) and CD8+ T cells (B). Ratios greater than one (dotted line) indicate enrichment of Ag-specific T cell events in the intrathecal compartment; ratios lower than one represent less pronounced intrathecal T cell responses. Horizontal bars represent median values; vertical lines represent interquartile ranges. (C) CD4+ T cell reactivities to foreign Ag's are shown for blood (upper panel) and CSF (lower panel) for the same patient cohorts described above. Box plots represent median values with 25th and 75th percentiles; black lines indicate minimum and maximum values. *0.01<p<0.05, **0.001<p<0.01, ***p<0.001. (TIF) [file pone.0105434.s004.tif]
